# Latent trajectory studies: the basics, how to interpret the results, and what to report

**DOI:** 10.3402/ejpt.v6.27514

**Published:** 2015-03-02

**Authors:** Rens van de Schoot

**Affiliations:** 1 Department of Methods and Statistics, Utrecht University, Utrecht, The Netherlands; 2 Optentia Research Program, Faculty of Humanities, North-West University, Mahikeng, South Africa

**Keywords:** Latent Growth Mixture Modeling, latent growth curve analysis, mixture modeling

## Abstract

**Background:**

In statistics, tools have been developed to estimate individual change over time. Also, the existence of latent trajectories, where individuals are captured by trajectories that are unobserved (latent), can be evaluated (Muthén & Muthén, 2000). The method used to evaluate such trajectories is called Latent Growth Mixture Modeling (LGMM) or Latent Class Growth Modeling (LCGA). The difference between the two models is whether variance within latent classes is allowed for (Jung & Wickrama, 2008). The default approach most often used when estimating such models begins with estimating a single cluster model, where only a single underlying group is presumed. Next, several additional models are estimated with an increasing number of clusters (latent groups or classes). For each of these models, the software is allowed to estimate all parameters without any restrictions. A final model is chosen based on model comparison tools, for example, using the BIC, the bootstrapped chi-square test, or the Lo-Mendell-Rubin test.

**Method:**

To ease the use of LGMM/LCGA step by step in this symposium (Van de Schoot, 2015) guidelines are presented which can be used for researchers applying the methods to longitudinal data, for example, the development of posttraumatic stress disorder (PTSD) after trauma (Depaoli, van de Schoot, van Loey, & Sijbrandij, 2015; Galatzer-Levy, 2015). The guidelines include how to use the software Mplus (Muthén & Muthén, 1998–2012) to run the set of models needed to answer the research question: how many latent classes exist in the data? The next step described in the guidelines is how to add covariates/predictors to predict class membership using the three-step approach (Vermunt, 2010). Lastly, it described what essentials to report in the paper.

**Conclusions:**

When applying LGMM/LCGA models for the first time, the guidelines presented can be used to guide what models to run and what to report.
